# Anterior wall STEMI in a patient with paroxysmal atrial fibrillation due to coronary embolism: A case report

**DOI:** 10.1016/j.amsu.2022.104602

**Published:** 2022-09-08

**Authors:** Newton Ashish Shah, Sangam Shah, Ashes Rijal, Anand Chaudhary, Swati Chand, Shailendra Pandey, Laba Rawal, Suraj Parajuli, Rajaram Khanal, Chandra Mani Poudel

**Affiliations:** a Tribhuvan University, Institute of Medicine, Maharajgunj, 44600, Nepal; b Kathmandu Medical College and Teaching Hospital, Kathmandu, Nepal; c Kathmandu University School of Medical Sciences, Dhulikhel, Nepal; d Department of Cardiology, Maharajgunj Medical Campus, Institute of Medicine, Tribhuvan University, Maharajgunj, 44600, Nepal; e Department of Cardiology, Manmohan Cardiothoracic Vascular and Transplant Center, Maharajgunj, 44600, Nepal

**Keywords:** Atrial fibrillation, STEMI, Coronary embolism

## Abstract

**Introduction:**

Coronary embolism (CE) is a rare cause of acute ST-elevation myocardial infarction (STEMI). Atrial fibrillation (AF), left ventricular thrombus, septic emboli from infective endocarditis, myxoma, and paradoxical embolism can induce emboli in coronary arteries.

**Case presentation:**

Here we present a case of anterior wall STEMI secondary to paroxysmal AF in a 60-years-old female with a previous history of right-sided ischemic stroke.

**Discussion:**

The major criteria for diagnosis of coronary embolism include (1) non-atherosclerotic wall of coronary vessels under angiography; (2) concomitant involvement of multiple sites; (3) histological proof of venous thrombus; (4) imaging by echocardiography/CT/MRI showing intra-cardiac thrombus. The minor criteria include (1) <25% stenosis of other vessels supplying to infarct-free myocardium; (2) atrial fibrillation history; (3) risk factors like (prosthetic valve, bacterial endocarditis, patent foramen ovale, atrial septal defect, dilated cardiomyopathy).

**Conclusion:**

Our case highlights the importance of cardiac embolus as a diagnosis in a patient with a history of stroke secondary to atrial fibrillation as a cause of acute STEMI and its management.

## Highlights

• Cardioembolism is an underappreciated etiology of the acute coronary syndrome in the patient with atrial fibrillation.
• Cardiac embolus may be in a patient with the history of stroke secondary to atrial fibrillation as a cause of acute STEMI.

## Introduction

Coronary embolism (CE) is a rare cause of acute ST-elevation myocardial infarction (ASTEMI). Atrial fibrillation (AF), left ventricular thrombus, septic emboli from infective endocarditis, myxoma, and paradoxical embolism due to a patent foramen ovale can all induce emboli to coronary arteries [1]. Paroxysmal AF is the most common cause of embolic stroke of undetermined cause. Patients who have had a stroke have an increased risk of recurrence as well as a higher risk of coronary embolism [2]. Here we present a case of a 60-years-old female with ASTEMI secondary to paroxysmal AF. This case has been reported as per SCARE 2020 criteria [3].

## Case presentation

A 60-years-old female presented to the emergency with a sudden onset of central chest pain that was sub-sternal, exertional, and non-radiating. She had no history of shortness of breath, loss of consciousness, orthopnea, paroxysmal nocturnal dyspnea (PND), palpitation, headache, vomiting, and fever. She didn't have hypertension, pulmonary tuberculosis, or a thyroid disorder. She did not smoke or consumed alcohol. She had right-sided ischemic stroke two years ago with minimal residual sensory and motor defects. She could ambulate by herself (modified Rankin Scale: 1). She was diagnosed with type II diabetes mellitus at the time of presentation.

On examination, her Glasgow coma scale (GCS) was (13/15). She was not oriented to time, place or person. Her blood pressure was 130/90 mm of Hg, pulse rate of 90 beats/minute, the body temperature of 98.5 °F, and respiratory rate of 18 breaths/minute. On examination, pallor, icterus, clubbing, cyanosis, or edema were absent. Her jugular venous pressure (JVP) was normal. On auscultation, the first and second heart sounds were heard without any murmur. Bilateral vesicular breath sounds were present.

Her laboratory findings revealed hemoglobin of 11 mg/dl (12–16 mg/dl), total leucocyte count 9060 cells/mm^3^ (4000–11000 cells/mm^3^), platelets 160000 cells/mm^3^ (150000–400000 cells/mm^3^), troponin I 0.83 ng/mL (<0.0004 ng/mL), creatinine kinase-MB (CK-MB) 59% of total CK (normal range: 3–5%), and HbA1C of 7.6% (normal range: <5.7%). The details of the laboratory investigations are shown in Table 1.

**Table 1:** Lab values of the patient.

Parameters	Values	Reference Range
Random blood sugar	140 mg/dl	<140 mg/dl
Urea	11 mg/dl	7–18 mg/dl
Creatinine	0.8 mg/dl	0.6–1.2 mg/dl
Sodium	139 mEq/L	136-146 mEq/L
Potassium	4.3 mEq/L	3,5-5 mEq/L
Triglyceride	56 mg/dl	<150 mg/dl
Cholesterol	118 mg/dl	<200 mg/dl
HDL	44 mg/dl	40–60 mg/dl
LDL	72 mg/dl	<160 mg/dl

**Fig. 1:**
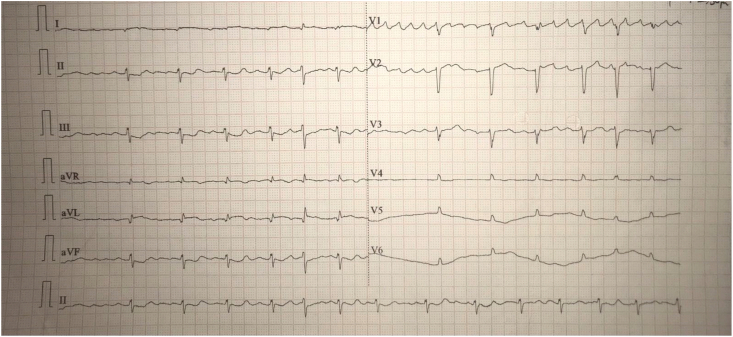
ECG showing anterior wall ST-elevation myocardial infarction.

ECG revealed anterior wall ST-elevation myocardial infarction (STEMI) (Fig. 1). The patient was managed with dual anti-platelets, statins, and diuretics. Evaluating the patient's stability, an emergency coronary angiography (CAG) was done which showed total thrombotic occlusion in the mid-left anterior descending artery (m-LAD) suggesting single vessel disease. Minor plaques were seen in the ostium of ramus intermedius (RI) and the ostium of LAD. In light of these discoveries, a suspicion of embolic myocardial infarction was done.

Percutaneous old balloon angioplasty (POBA) was done using non-compliant quantum [APEX 2 × 12 mm at 12 atmospheric pressures] and Apollo percutaneous transluminal coronary angioplasty (PTCA) balloon [2.5 × 10 mm at 12 atmospheric pressures]. Thrombosuction was done with a low-profile aspiration catheter kit and a significant amount of thrombus was aspirated.

Subsequently, she developed persistent bradycardia for which a 24-h HOLTER was placed which revealed sick sinus syndrome with intermittent 2:1 AV block and paroxysmal atrial fibrillation. There were multiple episodes of pauses (176) with the longest measuring 4.8 seconds. She was treated with oral anticoagulant (rivaroxaban 20 mg OD), antiplatelets (aspirin 75 mg OD and clopidogrel 75mg OD), statin (rosuvastatin 20 mg), and diuretics (20 mg BD) along with spironolactone (12.5mgOD). Metformin hydrochloride (500 mg BD) was started for the newly diagnosed type II diabetes mellitus. Hypokinesia of LAD territory, left ventricular ejection fraction of 30–35%, grade II left ventricular diastolic dysfunction, dilated left and right atrium, left ventricle, moderate mitral regurgitation, and tricuspid regurgitation (gradient:30 mmHg) were noted in echocardiography. Following this, she was discharged on oral medications after eleven days of hospital stay. She declined the suggestion of a permanent pacemaker. She was thoroughly counseled about possible complications of the condition (sudden cardiac death and syncopal attack) but she denied further treatment.

## Discussion

Plaque rupture, ulceration, fissure, erosion, or dissection of the thrombus in the artery, all are common causes of acute myocardial infarction. These can cause an imbalance in oxygen demand and supply in the heart, resulting in ischemic injury. Coronary artery embolism is a rare cause that results in 3% of acute coronary syndrome. The most common cause of direct coronary embolism is atrial fibrillation. In a study by Ruddox et al. it was established that AF heightens the risk of myocardial infarction with a relative risk of 1.84 without coronary artery disease [4]. Cardio embolism is an underappreciated etiology of acute coronary syndrome in patient with atrial fibrillation.

Atrial fibrillation is the most common cause of direct CE (73%) [5]. Direct CE originates from the aortic valves or the left atrial appendage [5]. Paroxysmal emboli originating from the venous circulation can cross through a patent foramen ovale in or during a cardiac intervention. Coronary embolism secondary to AF causing ACS (2.9%), 65% of which were STEMI, are considered rare complications [5]Thrombi from AF mostly get lodged in the cerebral or systemic circulation rather than coronary circulation. It may be because coronary arterial vasculature has relatively less caliber than bigger arteries like the aorta. In addition, the main arteries are at an acute angle at the origin of the aorta or there is a fast flow at the coronary ostia that changes the direction of the embolus [6].

Clinical presentation is no different from an atherosclerotic coronary stenosis. Regardless of the cause, the management is the same. But close follow-up and monitoring is required on patients with non-atherosclerotic MI than atherosclerotic MI as those patients are at higher risk of development of ischemia [7]. Patients with STEMI of AF origin suffer from a significant increased 5-year risk of thromboembolic cardiac and cerebrovascular events, which can be as high as 28% [5]. Recurrence of thromboembolic complication (10.4%) is another entity that should be considered which includes even recurrence of coronary embolism (4.2%) [5]. Risk factors like age (>60 years), female gender, reduced ejection fraction of the left ventricle, and atrial fibrillation can increase the risk for adverse non-sclerotic myocardial infarction [8].

In a study carried out by *Shibata* et al. [5], the major criteria for diagnosis of coronary embolism include (1) nonatherosclerotic wall of coronary vessels under angiography; (2)concomitant involvement of multiple sites; (3)histological proof of venous thrombus; (4) imaging by echocardiography/CT/MRI showing intra-cardiac thrombus. The minor criteria include (1) <25% stenosis of other vessels supplying to infarct-free myocardium; (2) atrial fibrillation history; (3) risk factors like (prosthetic valve, bacterial endocarditis, patent foramen ovale, atrial septal defect, dilated cardiomyopathy). The diagnostic criteria included the presence of ≥2 major criteria, 1 major and ≥2 minor criteria, or 3 minor criteria. Our patient met one major (angiography revealing thrombi in a non-atherosclerotic vessel) and two minor criteria (atrial fibrillation and <25% stenosis on other major coronary vessels) which qualified criteria for CE as the cause of STEMI [5]. Bacterial endocarditis was ruled out as the blood culture was non-reactive that supported non-vegetative thrombi lodged in the m-LAD. Paradoxical thrombi were ruled out as well because echocardiography did not show any breach in the interatrial or interventricular septum. In a *TAPAS* study on patients with acute myocardial infarction, thrombus aspiration has been shown to result in better reperfusion and clinical outcomes than conventional percutaneous coronary intervention (PCI)in patients with STEMI [9].

Patients with AF with a CHA_2_DS_2_VASc score of two in men and three in women should be treated with long-term anticoagulation. Our patient had a total score of 4 which showed a 4.8% per year unadjusted ischemic stroke occurrence rate [10]. The treatment recommended for CE is systemic anticoagulation. The mainstay of treatment is the prevention of the recurrence of thromboembolism. The risk of bleeding due to dual antiplatelet therapy and systemic anticoagulant should not be left unseen. In patients with bleeding risk, aspirin can be stopped for 4 weeks [11].

## Conclusion

Our case highlights the importance of recognizing cardiac embolus as a cause of acute STEMIand subseqeuent management in a patient with a history of stroke secondary to atrial fibrillation. Physicians should be aware of the clinical characteristics associated with CE in single vessel disease, its diagnostic evaluation, and future prevention of the risk of occurrence. Therapeutic challenges arise due to rare occurrences and late diagnosis of coronary embolism.

## Data availability statement

All the required information is within the manuscript itself.

## Provenance and peer review

Not commissioned, externally peer-reviewed.

## Ethical approval

None.

## Sources of funding

No funding was received for the study.

## Author contribution

NAS and SS prepared the original manuscript, reviewed, and edited the manuscript. SY, RK, and CMP reviewed and edited the manuscript. NAS, SS, AR, AC, SC, SP, LR, RC, SP, RK, CMP reviewed and edited the manuscript and were in charge of the case.

## Conflicts of interest

Authors have no conflict of interest to declare.

## Registration of research studies

1. Name of the registry: None
2. Unique Identifying number or registration ID: None
3. Hyperlink to your specific registration (must be publicly accessible and will be checked):

## Guarantor

Sangam Shah.

## Consent

Written informed consent was obtained from the patient for publication of this case report and accompanying images. A copy of the written consent is available for review by the Editor-in-Chief of this journal on request.

## References

[1] C C., Wr A. (2009). Acute myocardial infarction due to coronary artery embolism in a patient with atrial fibrillation. Neth. Heart J..

[2] Yd K., D S., Hs N., D C., Js K., Bk K., Hj C., Hy C., K L., J Y., Hs L., Cm N., Jh H. (2017). Increased risk of cardiovascular events in stroke patients who had not undergone evaluation for coronary artery disease. Yonsei Med. J..

[3] Agha R.A., Franchi T., Sohrabi C., Mathew G., Kerwan A., Thoma A., Beamish A.J., Noureldin A., Rao A., Vasudevan B., Challacombe B., Perakath B., Kirshtein B., Ekser B., Pramesh C.S., Laskin D.M., Machado-Aranda D., Miguel D., Pagano D., Millham F.H., Roy G., Kadioglu H., Nixon I.J., Mukhejree I., McCaul J.A., Chi-Yong Ngu J., Albrecht J., Rivas J.G., Raveendran K., Derbyshire L., Ather M.H., Thorat M.A., Valmasoni M., Bashashati M., Chalkoo M., Teo N.Z., Raison N., Muensterer O.J., Bradley P.J., Goel P., Pai P.S., Afifi R.Y., Rosin R.D., Coppola R., Klappenbach R., Wynn R., De Wilde R.L., Surani S., Giordano S., Massarut S., Raja S.G., Basu S., Enam S.A., Manning T.G., Cross T., Karanth V.K., Kasivisvanathan V., Mei Z. (2020). The SCARE 2020 guideline: updating consensus surgical CAse REport (SCARE) guidelines. Int. J. Surg..

[4] V R., I S., J M., J S., T E., Je O., Ruddox V., Sandven I., Munkhaugen J., Skattebu J., Edvardsen T., Otterstad J.E. (2017). Atrial fibrillation and the risk for myocardial infarction, all-cause mortality and heart failure: a systematic review and meta-analysis. Eur J Prev Cardiol.

[5] T S., S K., T N., T T., Y A., T K., T N., K N., M F., K N., H I.-U., K N., Y M., K K., T A., Y G., H O., S Y. (2015). Prevalence, clinical features, and prognosis of acute myocardial infarction attributable to coronary artery embolism. Circulation.

[6] Raphael C.E., Heit J.A., Reeder G.S., Bois M.C., Maleszewski J.J., Tilbury R.T., Holmes D.R.J., Ce R., Ja H., Gs R., Mc B., Jj M., Rt T., Dr H., Raphael C.E., Heit J.A., Reeder G.S., Bois M.C., Maleszewski J.J., Tilbury R.T., Holmes D.R.J. (2018). Coronary embolus: an underappreciated cause of acute coronary syndromes. JACC Cardiovasc. Interv..

[7] B P., N A., N B., S P., Ch M., Pa M., C S.S., Y J., E C. (2018). Coronary embolism among ST-segment-elevation myocardial infarction patients: mechanisms and management. Circ. Cardiovasc. Interv..

[8] Fa A., L L., Aq M., Y L., S X., R A., Y X., W C. (2019). Myocardial infarction with non-obstructive coronary arteries (MINOCA) in Chinese patients: clinical features, treatment and 1 year follow-up. Int. J. Cardiol..

[9] Vlaar P.J., Svilaas T., van der Horst I.C., Diercks G.F.H., Fokkema M.L., de Smet B.J.G.L., van den Heuvel A.F.M., Anthonio R.L., Jessurun G.A., Tan E.-S., Suurmeijer A.J.H., Zijlstra F. (2008). Cardiac death and reinfarction after 1 year in the Thrombus Aspiration during Percutaneous coronary intervention in Acute myocardial infarction Study (TAPAS): a 1-year follow-up study. Lancet (London, England).

[10] L F., M R., Gy L. (2012). Evaluation of risk stratification schemes for ischaemic stroke and bleeding in 182 678 patients with atrial fibrillation: the Swedish Atrial Fibrillation cohort study. Eur. Heart J..

[11] January C.T., Wann L.S., Alpert J.S., Calkins H., Cigarroa J.E., Cleveland J.C., Conti J.B., Ellinor P.T., Ezekowitz M.D., Field M.E., Murray K.T., Sacco R.L., Stevenson W.G., Tchou P.J., Tracy C.M., Anderson J.L., Halperin J.L., Albert N.M., Bozkurt B., Brindis R.G., Creager M.A., Curtis L.H., DeMets D., Guyton R.A., Hochman J.S., Kovacs R.J., Ohman E.M., Pressler S.J., Sellke F.W., Shen W.K., Yancy C.W. (2014). AHA/ACC/HRS guideline for the management of patients with atrial fibrillation: executive summary: a report of the American College of cardiology/American heart association task force on practice guidelines and the heart rhythm society. Circulation.

